# Effect of Plain Versus Sugar‐Sweetened Breakfast on Energy Balance and Metabolic Health: A Randomized Crossover Trial

**DOI:** 10.1002/oby.22757

**Published:** 2020-02-28

**Authors:** Harriet A. Carroll, Yung‐Chih Chen, Iain S. Templeman, Phoebe Wharton, Sue Reeves, William V. Trim, Enhad A. Chowdhury, Jeff M. Brunstrom, Peter J. Rogers, Dylan Thompson, Lewis J. James, Laura Johnson, James A. Betts

**Affiliations:** ^1^ Department for Health University of Bath Bath UK; ^2^ Rowett Institute University of Aberdeen Aberdeen UK; ^3^ Department of Physical Education National Taiwan Normal University Taipei Taiwan; ^4^ Institute for Research Excellence in Learning Science National Taiwan Normal University Taipei Taiwan; ^5^ Department of Life Sciences University of Roehampton London UK; ^6^ School of Psychological Science University of Bristol Bristol UK; ^7^ School of Sport, Exercise and Health Sciences Loughborough University Loughborough UK; ^8^ School for Policy Studies University of Bristol Bristol UK

## Abstract

**Objective:**

This study investigated the effect of 3 weeks of high‐sugar (“Sweet”) versus low‐sugar (“Plain”) breakfast on energy balance, metabolic health, and appetite.

**Methods:**

A total of 29 healthy adults (22 women) completed this randomized crossover study. Participants had pre‐ and postintervention appetite, health, and body mass outcomes measured, and they recorded diet, appetite (visual analogue scales), and physical activity for 8 days during each intervention. Interventions were 3 weeks of isoenergetic Sweet (30% by weight added sugar; average 32 g of sugar) versus Plain (no added sugar; average 8 g of sugar) porridge‐based breakfasts.

**Results:**

Pre‐ to postintervention changes in body mass were similar between Plain (Δ 0.1 kg; 95% CI: −0.3 to 0.5 kg) and Sweet (Δ 0.2 kg; 95% CI: −0.2 to 0.5 kg), as were pre‐ to postintervention changes for biomarkers of health (all *P* ≥ 0.101) and psychological appetite (all *P *≥ 0.152). Energy, fat, and protein intake was not statistically different between conditions. Total carbohydrate intake was higher during Sweet (287 ± 82 g/d vs. 256 ± 73 g/d; *P *= 0.009), driven more by higher sugar intake at breakfast (116 ± 46 g/d vs. 88 ± 38 g/d; *P* < 0.001) than post‐breakfast sugar intake (Sweet 84 ± 42 g/d vs. Plain 80 ± 37 g/d; *P* = 0.552). Participants reported reduced sweet desire immediately after Sweet but not Plain breakfasts (trial × time *P* < 0.001).

**Conclusions:**

Energy balance, health markers, and appetite did not respond differently to 3 weeks of high‐ or low‐sugar breakfasts.

**Study Importance**

**What is already known?**

► Sugar has been implicated in poorer metabolic health outcomes, positive energy balance, and appetite dysregulation, with the opposite true for breakfast.
► A recent review called for ecologically valid studies investigating sweetness with an adequate nonsweet comparator.
► Previous short‐term work found no effect of 5 days of high‐sugar breakfast on appetite.

**What does this study add?**

► This is the first study to investigate medium‐term (3 weeks) high‐ versus low‐sugar breakfast on physiological and psychological appetite and metabolic health.
► We found clear sensory‐specific satiety (reduced sweet desire) after the high‐sugar breakfast, but this did not impact energy or sugar intake.
► We found no change in any fasted markers of health or appetite.

## Introduction

Despite public health guidelines promoting breakfast consumption as a means to maintain weight 1, 2, previous research randomizing participants to consume or omit breakfast has found no effect on energy intake or weight, with a potential advantage of breakfast omission in reducing energy intake and body mass 3. Nonetheless, > 75% of the United Kingdom population consumes breakfast 4, 5, and the percentage of energy consumed from sugar is higher at breakfast than other eating occasions in the day 5. Accordingly, public health guidelines have recently recommended a reduction in breakfast sugar as a means to reduce energy intake and aid in maintaining metabolic health 6, 7.

Research has investigated known satiating nutrients, such as fiber‐ or protein‐enriched breakfasts, finding these to mitigate multiple aspects of appetite 8, 9, 10. Yet, despite typical breakfasts being high in sugar, to our knowledge, there is no causal evidence directly implicating high‐sugar breakfasts in appetite dysregulation or associated detrimental health outcomes. One study investigated the effect of 5 days of isoenergetic plain versus sugar‐sweetened versus artificially sweetened breakfasts on hourly hunger ratings and food intake in 24 healthy adults 11. Overall, there was no effect on hunger or energy intake between the plain and sugar‐sweetened (15.7% by weight sugar) breakfasts. However, this short‐term study did not measure physical activity, changes in appetite, or markers of metabolic health.

Thus, we aimed to compare the effects of a high‐sugar (“Sweet”) versus no‐added‐sugar (“Plain”) breakfast on energy balance and metabolic health markers in healthy adults. We first hypothesized that a calorically sweetened breakfast would reduce post‐breakfast sweet desire because of sensory‐specific satiety 12, leading to lower net daily energy intake and subsequent weight loss (primary outcome) compared with a plain breakfast. Second, we hypothesized that, because of sugar‐stimulated glucagon‐like peptide‐1 (GLP‐1) secretion 13, fasting markers of glucose regulation would improve after the sugar‐sweetened porridge. Thirdly, because sugars are implicated in increased circulating triacylglycerol (TAG) compared with starches 14, we also anticipated finding an increase in fasting TAG concentrations after the Sweet versus Plain breakfast, which may be detrimental to cardiometabolic health.

## Methods

### Participants

**Figure 1:**
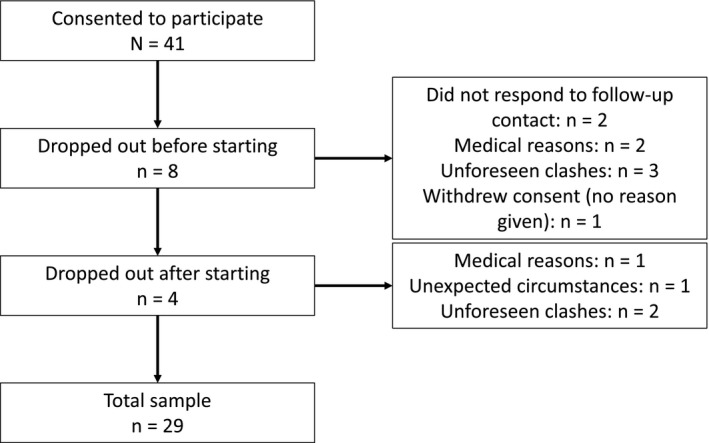
Flowchart of participant retention.

A total of 41 participants gave informed consent to participate in this study, with 29 (22 women) completing the study and being included in the analyses (Figure 1). Mean (SD) age and BMI of those who completed the protocol were 33 (10) years and 25.0 (5.2) kg∙m^−2^, respectively. Randomization strata were based on sex and weight status (BMI‐determined healthy [< 25 kg/m^2^] vs. BMI‐determined overweight/obesity [≥ 25 kg/m^2^]).

Sample size was estimated based on 80% power with α = 0.05, targeting estimated energy intake reduction (225 kcal/d) resulting in body mass loss (656 g) based on data from the Bath Breakfast Project 15. See online Supporting Information for details on randomization, sample size estimation, and design rationale. Exclusion criteria (self‐reported) were as follows: being age < 18 years or ≥ 70 years, having metabolic disease (no body mass restrictions, except self‐reported weight loss > 5 kg in previous 6 months), having drug dependency (excluding caffeine and nicotine), being pregnant or breastfeeding, and using a medication that may interact with the intervention to cause harm or introduce bias; thus all participants were considered metabolically healthy (though *n* = 11 with BMI‐defined overweight/obesity). Data were collected in South West England between May 2017 and October 2018, inclusive (trial registration: osf.io/hbn7s; deviations to the protocol are explained throughout). Ethical approval was obtained from the Research Ethics Approval Committee for Health at the University of Bath (reference: EP 16/17 166), and the study was conducted in accordance with the Declaration of Helsinki.

### Study design

**Figure 2:**
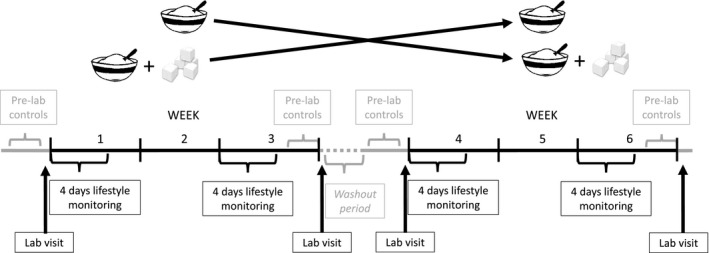
Schematic of the trial protocol.

This was a participant‐blinded (i.e., intention‐masked) randomized crossover study, with a 4‐ to 60‐day washout (mode: 6 days; median: 16 days [interquartile range: 6‐32]). The washout period was extended from our registered protocol because of unforeseen circumstances (e.g., snow) causing delays, which had to be accommodated when controlling for the menstrual cycle. To reduce confounding in fasted measures of health and appetite taken in the laboratory, each trial arm consisted of 3 days of replicating physical activity and 1 day of replicating diet (“pretesting control”) that were standardized between trials (i.e., within participant) (details in online Supporting Information). The study then consisted of a pre‐ and postintervention laboratory visit for fasted measures and two 3‐week free‐living Plain or isoenergetic Sweet breakfast interventions (detailed subsequently and in Figure 2).

### Protocol

#### Laboratory testing: anthropometrics and blood pressure

Participants arrived at the laboratory between 0600 and 0900 hours fasted, standardized between visits by ± 1 hour. A urine sample was provided, followed by measurement of body mass, measured to the nearest 0.1 kg in duplicate (or triplicate if measures differed by ≥ 0.1 kg) (InnerScan, Model BC‐543, Tanita Corp.), and height, recorded using a stadiometer to the nearest 0.1 cm (Seca 222). Duplicate (or triplicate if measures differed by > 0.3 cm) measures of hip circumference (widest gluteal girth when standing) and waist circumference (midpoint between lowest rib and iliac crest) (Seca 201 measuring tape) were then taken 16. Blood pressure was measured in duplicate (or triplicate if measures differed by ≥ 3 mmHg) on the left arm with an automatic sphygmomanometer (Panasonic EW3106W).

#### Laboratory testing: blood sampling

A 10‐mL blood sample was then taken from an antecubital vein while the participant lay semisupine. Blood was collected into an ethylenediaminetetraacetic‐acid‐coated tube (BD) for plasma and a serum tube for serum (BD). Serum was left to clot for ~30 minutes at room temperature before being centrifuged. Samples were centrifuged at 3,446*g* for 10 minutes at 4°C, and the supernatant was immediately frozen at −20°C before being moved to a −70°C freezer for longer‐term storage.

#### Laboratory testing: appetite measures

In silence, participants completed a range of paper‐based questionnaires assessing various aspects of their appetite. A visual analogue scale (VAS) asked “How hungry/full/thirsty do you feel?”, “How much food do you think you can eat?”, and “How strong is your desire to eat something sweet/savory/salty/fatty?” Participants were instructed to place a vertical line corresponding to their feeling on a 100‐mm horizontal scale, anchored between two extremes, with “not at all” (or equivalent) to the left (scored as 0) and “extremely” (or equivalent) to the right (scored as 100). On the two postintervention laboratory visits, participants additionally used a VAS to rate how sweet, salty, savory, and fatty they thought the porridge was during the intervention.

Following the VAS, participants completed the 39‐item Trait Version and 15‐item State Version Food Craving Questionnaires 17, the Food Craving Inventory 18, the Morningness‐Eveningness Questionnaire 19, and the Three‐Factor Eating Questionnaire 20. The Food Craving Inventory was modified to make it more applicable to British norms (e.g., “French fries” was changed to “chips,” and “chips” was changed to “crisps”). Our trial registration included a forced‐choice computer task, which will be used for pilot data (data have not been presented herein).

#### Laboratory testing: energy expenditure

Resting metabolic rate was measured via indirect calorimetry from gaseous exchange 21, whereby the average of four 5‐minute expired gas samples was taken. Samples were collected in a Douglas bag (Hans Rudolph), with participants in a semisupine position after ≥ 30 minutes of rest. Inspired air composition was measured simultaneously to adjust for ambient conditions 22. Gas concentrations were measured using paramagnetic and infrared analyzers (Mini HF 5200, Servomex Group Ltd.). Two participants were excluded from analysis of resting metabolic rate and respiratory exchange ratio because a leak in the Douglas bag gave invalid data.

#### Laboratory testing: intervention

The intervention consisted of two 3‐week periods of consuming either a Plain or a Sweet porridge breakfast (Lidl Oatilicious Scottish Porridge Oats), provided in a random order. The breakfasts were isoenergetic; thus, the Sweet breakfast had fewer oats and it was 30% castor sugar by dry weight (Sainsbury’s White Caster Sugar), based on the upper end of the average sugar content of 101 UK breakfast cereals (Supporting Information Table S4). Participants added their usual amount of milk in the laboratory; the weight of this was noted, and participants were instructed to add this amount for the entirety of the study (compliance assessed via self‐completed breakfast log). Including the milk added by participants, average breakfast compositions were as follows (Plain vs. Sweet): energy: 1,653 (343) kJ vs. 1,637 (347) kJ; energy density: 1.3 (0.2) kcal/g vs. 1.3 (0.2) kcal/g; carbohydrates: 58 (10) g vs. 67 (11) g; fiber: 6 (1) g vs. 4 (1) g; sugar: 8 (6) g vs. 32 (7) g; fat: 11 (3) g vs. 8 (3) g; saturated fat: 3 (2) g vs. 3 (2) g; protein: 15 (4) g vs. 12 (4) g; and water: 214 (61) g vs. 210 (61) g (further details given in online Supporting Information).

Porridge prepackaged into one‐breakfast portions was provided to participants. Participants were instructed that the full amount of porridge should be consumed as the first energy ingested within 2 hours of waking (consistent with a recent definition of breakfast  23) and that no energy should be consumed for 1 hour after eating the porridge, after which ad libitum intake was allowed. Black/green tea, black coffee, and water were allowed before, during, and after breakfast. Minimum compliance was consuming the porridge as instructed ≥ 5 d/wk (assessed via breakfast log), which all 29 participants achieved.

During days 1 to 4 and 15 to 18 in each 3‐week period, participants had their physical activity measured (ActiHeart, CamNtech), and they recorded their diet (weighed food records) (details in online Supporting Information) and appetite (paper‐based VAS). The eight appetite VASs given in the laboratory were given to participants to be completed before and after breakfast, before self‐defined lunch, and before self‐defined dinner. Other than the rules surrounding the breakfast, no other dietary/lifestyle instructions were given.

### Biochemical analysis

Analytes were measured using commercially available enzyme‐linked immunosorbent assays (serum insulin and plasma GLP‐1), spectrophotometric assays (plasma glucose and total cholesterol), freezing‐point depression (serum osmolality), and electrochemiluminescence (fibroblast growth factor 21 [FGF21]). Urine specific gravity was measured using a handheld refractometer. We were unable to obtain a blood sample for every participant; thus sample size was 24 for all blood measures except FGF21 (*n* = 13; details in online Supporting Information).

### Statistical analysis

For measures taken in the laboratory (e.g., body mass), a change score was calculated (postintervention value minus preintervention value), and paired‐samples *t* tests were conducted comparing the change scores between Plain and Sweet. For relevant variables during the intervention, the differences between the mean in Plain and Sweet were assessed using a paired‐samples *t* test. VASs completed throughout the interventions were analyzed via repeated‐measures ANOVA comparing the average of the 8 days of VAS recording for each intervention. Order effects were tested by rerunning the analyses with trial order included as a covariate. Analyses were also rerun with the inclusion of BMI as a covariate; this did not meaningfully change any of our findings and therefore has not been discussed further.

Normality was checked visually using normal and detrended normal standardized P‐P and Q‐Q plots of residuals. Appropriate nonparametric equivalents were used for non‐normal data. For repeated‐measures ANOVA, asphericity was assessed via the Greenhouse‐Geisser epsilon, with the Greenhouse‐Geisser or Huynh‐Feldt correction being applied for values < 0.75 and > 0.75, respectively. Data are presented as mean (SD) or mean and 95% confidence interval (CI) (normalized to account for between‐participant variation). Data were analyzed using SPSS Statistics software version 22 (IBM Corp.).

## Results

Out of 59 outcomes, 9 (fasted hunger and salt desire [VAS]; Morningness‐Eveningness Questionnaire; hunger [Three‐Factor Eating Questionnaire]; plasma cholesterol concentration; during‐intervention fullness; how much one can eat; sweet desire and fatty desire [VAS]) had evidence of an order effect, though this did not appear to meaningfully alter our results (Supporting Information Table S1; discussed in online Supporting Information).

### Pre‐ to postintervention changes: anthropometrics and health markers

Participants started each intervention with similar health markers (Table 1 and Supporting Information Table S2). Body mass remained stable during both the Plain (Δ pre to post intervention 0.1 kg; 95% CI: −0.3 to 0.5 kg) and Sweet (Δ 0.2 kg; 95% CI: −0.2 to 0.5 kg) interventions; thus, we were unable to reject the null hypothesis of no differences in body mass change between conditions (*P* for difference in change = 0.780; Table 1). Equally, the null hypothesis was not rejected for change in BMI and waist‐hip ratio between trial arms (*P* for difference in change = 0.786 and 0.935, respectively) (Table 1).

**Table 1:** Anthropometric and health markers, pre to post, for each intervention (*n* = 29)

	Plain			Sweet					
	Pre (mean ± SD)	Post (mean ± SD)	Δ (95% CI), pre to post	Pre (mean ± SD)	Post (mean ± SD)	Δ (95% CI), pre to post	*P*_diff_ pre, Plain vs. Sweet	*P*_diff_ post, Plain vs. Sweet	*P*_diff_ Δ, Plain vs. Sweet
**Body mass (kg)**	72.6 ± 15.0	72.7 ± 15.0	0.1 (−0.3 to 0.5)	72.3 ± 15.2	72.4 ± 15.0	0.2 (−0.2 to 0.5)	0.087	0.190	0.780
**BMI (kg∙m^−2^)**	25.1 ± 5.2	25.1 ± 5.2	0.0 (−0.1 to 0.2)	24.9 ± 5.3	25.0 ± 5.2	0.1 (−0.1 to 0.2)	0.146[Link]	0.118[Link]	0.786
**Waist‐hip ratio**	0.79 ± 0.07	0.79 ± 0.07	0.00 (−0.01 to 0.01)	0.79 ± 0.07	0.79 ± 0.08	0.00 (−0.02 to 0.01)	1.000	0.679	0.935[Link]
**Glucose (mmol∙L^−1^)**	5.4 ± 0.4	5.4 ± 0.4	0.0 (−0.1 to 0.1)	5.4 ± 0.4	5.5 ± 0.4	0.1 (−0.1 to 0.2)	0.926	0.369	0.819[Link]
**Cholesterol (mmol∙L^−1^)**	4.45 ± 0.93	4.26 ± 0.69	−0.19 (−0.38 to 0.00)	4.27 ± 0.86	4.31 ± 0.70	0.04 (−0.14 to 0.22)	0.067	0.581	0.194[Link]
**Insulin (pmol∙L^−1^)**	34.73 ± 26.79	35.55 ± 23.31	0.82 (−6.29 to 7.93)	35.83 ± 19.10	42.70 ± 21.40	6.86 (−2.44 to 16.16)	0.884[Link]	0.137[Link]	0.278[Link]
**HOMA‐IR**	1.2 ± 0.9	1.2 ± 0.8	0.0 (−1.2 to 0.3)	1.2 ± 0.7	1.5 ± 0.8	0.3 (−0.1 to 0.6)	0.658	0.081	0.288
**TAG (mmol∙L^−1^)**	0.87 ± 0.48	0.98 ± 0.56	0.10 (−0.04 to 0.24)	0.88 ± 0.35	0.92 ± 0.40	0.04 (−0.05 to 0.12)	0.784[Link]	0.578[Link]	0.136[Link]
**GLP‐1 (pmol∙L^−1^)**	17.3 ± 11.7	19.7 ± 10.3	2.4 (−2.2 to 7.0)	16.5 ± 9.7	17.9 ± 8.1	1.3 (−1.6 to 4.3)	0.654	0.401	0.702
**FGF21 (pg∙mL^−1^)**	879.5 ± 737.1	986.0 ± 871.9	106.5 (−127.7 to 340.7)	824.6 ± 771.0	832.1 ± 685.1	7.5 (−166.9 to 182.0)	0.701[Link]	0.101[Link]	0.500

All blood analytes are plasma concentrations, except insulin, which is serum concentration. All blood measures are concentrations in plasma, except insulin, which was measured from serum.* n* = 29 for body mass, BMI, waist‐hip ratio; *n* = 24 for glucose, cholesterol, TAG, insulin, GLP‐1; *n* = 13 for FGF21.

Data analyzed using Wilcoxon signed rank test. All other data analyzed using paired‐samples *t* test.

FGF21, fibroblast growth factor 21; GLP‐1, glucagon‐like peptide‐1; HOMA‐IR, homeostatic model assessment of insulin resistance; TAG, triacylglycerol.

#### Metabolism

The null hypothesis of no change between trial arms was not rejected for resting metabolic rate and respiratory exchange ratio (all *P* ≥ 0.257; Supporting Information Table S2). Similarly, we failed to reject the null hypothesis of no change from pre‐ to postintervention for fasted plasma glucose, TAG, FGF21, GLP‐1, serum insulin concentrations, homeostatic model assessment of insulin resistance, or serum osmolality (all *P* ≥ 0.136; Table 1). Plasma total cholesterol concentration decreased by 0.19 mmol/L (95% CI: −0.38 to 0.00) after the Plain intervention and had no change after the Sweet intervention (Δ 0.04 mmol/L; 95% CI: −0.14 to 0.22 mmol/L), with the null hypothesis unable to be rejected for pre‐ to postintervention change between Plain and Sweet (*P* for difference in change = 0.194; see online Supporting Information for discussion).

#### Appetite

From the VASs, the null hypothesis of no change across or between interventions was unable to be rejected (Supporting Information Table S3). There was a small increase in fasted hunger after the Sweet intervention (Δ 11 mm; 95% CI: 1 to 20 mm), but this change appeared similar to that of the Plain intervention (Δ 3 mm; 95% CI: −6 to 11 mm; *P* for difference in change = 0.221). Furthermore, after the Sweet intervention, there was a small reduction in desire for both sweet (Δ −8 mm; 95% CI: −17 to 0 mm) and savory (Δ −10 mm; 95% CI: −17 to −4 mm), but we were unable to reject the null hypothesis for the difference in these changes (*P* for difference in change ≥ 0.152; Supporting Information Figure S1 and Table S3; see online Supporting Information for order effects discussion).

Neither trait (*P* = 0.885) nor state food cravings changed differently between interventions (*P* ≥ 0.766). Similarly, the null hypothesis was not rejected for change in restraint, disinhibition, or hunger from the Three‐Factor Eating Questionnaire (all *P* ≥ 0.141). The Food Craving Inventory assessed desire for high fats, sweets, carbohydrates/starches, and fast‐food fats; again we were unable to reject the null in change across interventions (all *P* ≥ 0.253; Supporting Information Table S3; see online Supporting Information for order effects discussion).

### Lifestyle monitoring during each intervention

#### Diet

**Figure 3:**
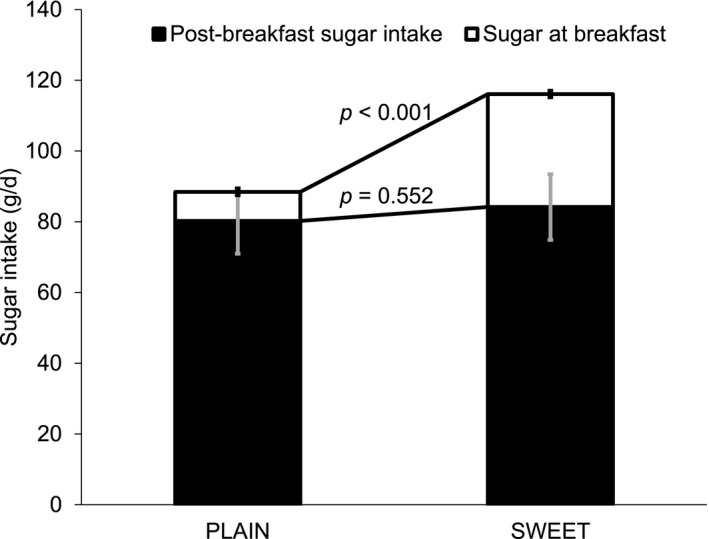
Total and post‐breakfast daily sugar intake according to breakfast intervention (*n* = 29). Error bars: normalized confidence intervals.

We were unable to reject the null hypothesis of no change for average self‐reported total daily energy intake across 8 days of diet recording during each of the interventions (Plain 9,393 [SD 2,507] kJ/d vs. Sweet 9,908 [SD 2,754] kJ/d; *P* = 0.112; Table 2). Total self‐reported daily carbohydrate intake was higher during Sweet (287 [SD 82] g/d) compared with Plain (256 [73] g/d; *P* = 0.009). The greater carbohydrate intake fully accounts for the slightly higher energy intake between Plain and Sweet, and it was driven by higher sugar prescribed during Sweet (116 [SD 46] g/d) compared with Plain (88 [SD 38] g/d; *P* < 0.001). The difference in sugar intake between conditions was solely due to the sugar given at breakfast (post‐breakfast sugar intake: Plain 80 [SD 37] g/d vs. Sweet 84 [SD 42] g/d; *P* = 0.552; Table 2; Figure 3; Supporting Information Figure S2 shows individual data).

**Table 2:** Diet and physical activity during each intervention (*n* = 29)

	Plain (mean ± SD)	Sweet (mean ± SD)	*P*_difference_, Plain vs. Sweet
**Energy intake (kJ∙d^−1^)**	9,393 ± 2,507	9,908 ± 2,754	0.112
**Carbohydrates (g∙d^−1^)**	256 ± 73	287 ± 82	0.009
**Sugar (g∙d^−1^)**	88 ± 38	116 ± 46	< 0.001[Link]
**Post‐breakfast sugar (g∙d^−1^)**	80 ± 37	84 ± 42	0.552
**Fiber (g∙d^−1^)**	26 ± 10	25 ± 10	0.170
**Fat (g∙d^−1^)**	85 ± 26	85 ± 27	0.804
**Saturated fat (g∙d^−1^)**	29 ± 12	29 ± 12	0.984[Link]
**Protein (g∙d^−1^)**	95 ± 36	97 ± 35	0.294
**Total water (food + fluid; g∙d^−1^)**	2,888 ± 1,405	2,956 ± 1,449	0.642
**Physical activity EE (kJ∙d^‐1^)**	3,202 ± 1,553	3,127 ± 1,599	0.820
**EE sedentary (kJ∙d^−1^)**	511 ± 188	569 ± 373	0.462
**EE light (kJ∙d^−1^)**	996 ± 393	913 ± 331	0.094
**EE moderate (kJ∙d^−1^)**	1,168 ± 846	1,147 ± 963	0.721
**EE vigorous (kJ∙d^−1^)**	327 ± 306	339 ± 335	0.665

Analyzed using paired‐samples *t* test. All other data analyzed using Wilcoxon signed rank test.

EE, energy expenditure.

#### Physical activity energy expenditure

The null hypothesis of no change was unable to be rejected for physical activity energy expenditure between Plain (3,202 [SD 1,553] kJ/d) and Sweet (3,127 [SD 1,599] kJ/d; *P* = 0.820; Table 2) or for intensities of physical activity (all *P* ≥ 0.094; Table 2).

#### Appetite

Participants were not told what the intervention was and so were asked to rate the flavors of the breakfast after each intervention. The Sweet breakfast was rated as being sweeter (87 [10] mm vs. Plain 15 [19] mm; *P* < 0.001) and less savory (23 [28] mm vs. Plain 47 [35] mm; *P* < 0.001). We were unable to reject the null hypothesis in ratings of saltiness (*P* = 0.129) or fattiness (*P* = 0.875). Liking of the breakfasts was similar between Plain (44 [24] mm) and Sweet (48 [28] mm) (*P* = 0.433).

**Figure 4:**
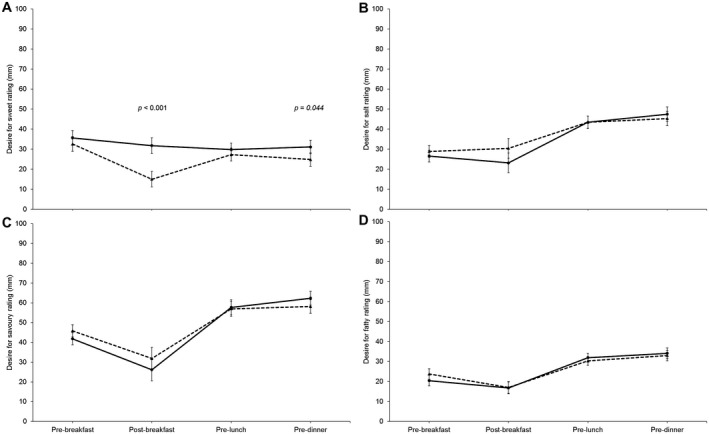
Visual analogue scale ratings for desire for (**A**) sweet, (**B**) salty, (**C**) savory, and (**D**) fatty across the day (*n* = 29). Dashed lines are “Sweet,” and full lines are “Plain.” Error bars: normalized confidence intervals. *P* values represent comparison of Plain vs. Sweet for that time point and are adjusted (Bonferroni) for multiple comparisons. Only significant *P* values are shown.

VASs (Figure 4 and Supporting Information Figure S3) throughout the day showed that there was a significant time effect for all variables (all *P* ≤ 0.001) except thirst (time *F* = 2.407, *P* = 0.112; Supporting Information Figure S3d). We were unable to reject the null for trial differences between Plain and Sweet for any variables (all *P* ≥ 0.082) except desire for sweet (trial *F* = 3538.317, *P* < 0.001) and fullness (trial *F* = 7.790, *P* = 0.010). There was a significant time × trial effect for how much participants felt they could eat (*F* = 4.060, *P* = 0.014) and desire for sweet (*F* = 12.280, *P* < 0.001), salt (*F* = 4.460, *P* = 0.008), and savory (*F* = 3.713, *P* = 0.037) (Figure 4 and Supporting Information Figure S3). Desire for sweet was similar between trial arms before breakfast and lunch but slightly lower during Sweet before dinner (*P* = 0.044). Post breakfast, however, desire for sweet was lower (−17 mm; *P* < 0.001) after the Sweet breakfast versus Plain (Figure 4; see online Supporting Information for order effects discussion).

## Discussion

This randomized crossover trial is, to our knowledge, the first to directly investigate the causal role of sugar at breakfast on metabolic health and appetite. We were unable to reject the null hypothesis of no change between a sugar‐sweetened and isoenergetic plain porridge breakfast over 3 weeks for fasted markers of metabolic health or most facets of appetite. As hypothesized, Sweet mitigated post‐breakfast sweet desire. However, this resulted in neither lower total daily energy intake nor weight change compared with Plain. Our second hypothesis, that fasting markers of glucose regulation would improve during Sweet, was rejected, as both glucose and insulin concentrations remained similar across both interventions, as did TAG concentrations. Discussion points are further considered in online Supporting Information.

We failed to reject the null hypothesis of no change in our primary outcome of body mass between trial arms. Considering that we failed to reject the null hypothesis for energy intake and physical activity energy expenditure between Plain and Sweet, it is unsurprising that weight change between interventions was not different. To our knowledge, this is the first study to measure free‐living energy balance behaviors in relation to manipulating sugar intake at breakfast in humans. These findings support previous shorter‐term research showing no impact on hunger or energy intake when consuming a plain versus high‐sugar breakfast for 5 days 11. Furthermore, our results suggest that the higher morning physical activity found after consuming compared with skipping breakfast 15 is likely related to the absence of energy, rather than the carbohydrate composition of breakfast per se, as we were unable to reject the null in total daily physical activity (though time effects should be further explored).

During the interventions, the null hypothesis was unable to be rejected for ratings of hunger and satiety between Plain and Sweet across the day. Melanson et al. 24 found a rapid versus extended blood glucose response to be associated with greater immediate satiety but also greater hunger before the next meal. Although we did not test postprandial glucose concentrations, we can assume that the Sweet breakfast caused a more rapid glycemic response because of its higher sugar content. However, hunger and fullness ratings were still similar across the day between conditions. Thus, we did not find evidence that a high dose of sugar had an appetite stimulatory effect at subsequent meals, concordant with previous research 25.

The reduction in sweet desire immediately after breakfast is consistent with literature on sensory‐specific satiety 12. Despite the reduction in post‐breakfast sweet desire, daily sugar intake was higher during Sweet compared with Plain. This difference is fully accounted for by the sugar provided in the Sweet breakfast. These findings appear to support public health guidelines that advise reducing breakfast sugar in order to reduce total daily sugar, since post‐breakfast sugar intake was similar between conditions. Thus, consumption of a high‐sugar breakfast did not prime participants to consume more sugar later in the day nor did it reduce intake, discordant with previous research 26, 27.

Typically, sensory‐specific satiety does not cause a reduction in pleasantness for other sensory stimuli 12. It is therefore surprising that the Sweet breakfast induced a reduction in desire for savory that was of a similar magnitude to the reduction found after consuming the Plain breakfast. As the breakfast was porridge based, we may have satiated desires for both sweet and savory simultaneously, for example, due to the association of porridge with savory flavors. Alternatively, the habit of consuming sweet after savory food, rather than vice versa, may have played a role; i.e., consumption of sweet food may signal the end of a meal, thereby reducing desire for both sweet and savory. Such ideas should be tested in future research.

Neither participants’ “hunger,” “restraint,” and “disinhibition” (measured by the Three‐Factor Eating Questionnaire) nor their desire for high fat, sweets, carbohydrates/starches, or fast‐food fats (measured by the Food Craving Inventory) caused us to reject the null hypothesis pre to post intervention between trials. Thus, repeated exposure to a sweet breakfast does not appear to impact perceived control over appetite or cravings. Such results are concordant with the similar GLP‐1 and FGF21 concentrations, both of which have been implicated in reducing food reward and sweet intake 28, 29. However, we did not measure postprandial hormone or psychological responses to the breakfasts under controlled conditions, which may have presented differences in appetite, GLP‐1, and/or FGF21.

Additionally, we were unable to reject the null hypothesis for fasted biomarkers of metabolic health or appetite, possibly because of the lack of change in energy balance behaviors. One explanation may be the lower postprandial glucose response experienced in the morning versus evening 30. Thus, our null findings for glucoregulatory markers could be because feeding sugar specifically at breakfast may have reduced the likelihood of observing detrimental health effects from sugar ingestion due to greater glucose tolerance in the morning.

Because previous research has shown an increase in TAG after sugar ingestion 31, 32, 33, we anticipated an increase after Sweet that was not found, supporting evidence that a high intake of sugar is needed in order to raise fasting TAG concentrations 14, 34. Because the sugar content we provided at breakfast was based upon the upper end of the typical sugar content found in commonly consumed breakfast cereals, our results are likely more applicable to real‐world eating habits compared with studies that have used high sugar loads.

This study has helped to fill several gaps recently identified in the literature, such as the need for research to be relevant to public health, to include a clear nonsweet comparator, to fully assess diet, and the to include a wider array of psychological measures 35. However, several outstanding areas for future research remain. First, we matched breakfasts for energy content in order to reduce the confounding effects of differences in energy sensing; however, in free‐living conditions, people may add (rather than substitute) sugar to their cereal, thus altering energy balance. Accordingly, our findings need to be taken in the context of a fixed‐energy breakfast; a sweet breakfast may induce polyphagia if ad libitum breakfast intake was allowed and there was no post‐breakfast food intake restriction. Second, although we have substantially built on previous literature using a 21‐day intervention, future work should measure longer‐term responses as per recent suggestions 35. Thirdly, porridge is high in fiber and is typically consumed warm, both of which can affect appetitive and metabolic health outcomes. Thus, different types of cereals and breakfasts should be investigated.

Overall, this study found no evidence that 3 weeks of a high‐sugar porridge breakfast causes detrimental effects to fasted markers of metabolic health, dysregulates energy balance, or stimulates appetite compared with an isoenergetic plain porridge breakfast. We found that a high‐sugar breakfast contributed to higher daily (but not post‐breakfast) sugar intake. Therefore, reducing sugar at breakfast might be a viable way to reduce total daily sugar intake as per current public health recommendations.

## Funding agencies

This work was supported by the Economic and Social Research Council (grant no. ES/J50015X/1). The Economic and Social Research Council had no role in the design, analysis, or writing of this article.

## Disclosure

HAC has accepted conference fees from Danone Nutricia Research and a Graduate Research Grant for unrelated work from the European Hydration Institute. JAB has received research funding from Biotechnology and Biological Sciences Research Council (BBSRC), GlaxoSmithKline, Lucozade Ribena Suntory, Kellogg’s, Nestlé, and PepsiCo and is a scientific advisor to the International Life Sciences Institute (ILSI). PJR has received funding for research from Sugar Nutrition UK, has provided consultancy services for Coca‐Cola Great Britain, and is a scientific advisor to the International Life Sciences Institute‐Europe. LJ has had research funding from Danone Baby Nutrition, the Alpro Foundation, Kellogg Europe, and various UK research councils. DT has received both funding and consultancy fees from Unilever. LJJ has received funding from PepsiCo, Volac International, The Collagen Research Institute, British Summer Fruits, and Engineering and Physical Sciences Research Council (EPSRC). SR has received research funding from Kellogg’s and Dr. Schar. The other authors declared no conflict of interest.

## Author contributions

Study conception: HAC, JMB, PJR, LJJ, LJ, and JAB; data analysis: HAC, YCC, IST, PW, SR, WVT, and EAC; data collection: HAC, YCC, and IST; supervision: DT, LJJ, LJ, and JAB; drafted manuscript: HAC. All authors approved the submitted version.

## Trial registration

OSF: osf.io/hbn7s (nonclinical trial).
