# Supplementary material for: Effect of Plain Versus Sugar‐Sweetened Breakfast on Energy Balance and Metabolic Health: A Randomized Crossover Trial
Source: Obesity (Silver Spring). 2020 Feb 28;28(4):740–8. doi: 10.1002/oby.22757 (PMC7154643; doi:10.1002/oby.22757)
Supplement: Supplementary file 1 [file OBY-28-740-s001.docx]

# Supplementary Material Subsection 1: Methodological details

### Randomisation

Randomisation was conducted by a technician who was not part of the research team, using www.randomization.com. Block sizes were 4-6 in size, and stratifications were made based on binary sex (male/female) and BMI (BMI-healthy/BMI-overweight using the cut-off as </≥ 25 kg∙m^-2^). These strata were decided upon due to potential sex differences in energy balance compensatory mechanisms (1, 2), and differences in sweet perception and GLP-1 sensitivity related to weight status.

The average BMI of those classed as ‘BMI-healthy’ was 22.0 ± 1.6 kg∙m-2 (n = 18; 15 women), compared to 30.0 ± 5.3 kg∙m-2 (n = 11; 7 women) for those in the ‘BMI-overweight’ category. Sixteen participants were randomized to the PLAIN intervention first; in the ‘BMI-healthy’ cohort, nine (2 male) were randomized to PLAIN first, compared to 7 (3 male) in the ‘BMI-overweight’ cohort.

### Sample size estimation

The sample size estimate was based on detecting the smallest worthwhile effect of an energy intake reduction of 225 kcal/d, leading to an approximate body mass loss of 656 g over the three week intervention. Based on data from the Bath Breakfast Project (3), n = 14 participants in the lifestyle maintenance group (i.e. no intervention for six weeks), average body mass change was -90 g with a standard deviation of 1 100 g. Thus for this study, body mass change of 90 g vs 656 g with a pooled standard deviation of 1 100 g = effect size (*dz*) of 0.51. With an alpha of 0.05 and a beta of 0.8 using a two tailed t-test, we aimed to have 32 participants complete the study (calculated using G*Power). Weight loss of the predicted magnitude has previously been found in a three week intervention manipulating foods with different hedonic values (4).

We had not initially planned to measure FGF21 but were able to due to leftover kits from another project, hence the reduction in sample size for this hormone. Participants were randomly selected for FGF21 analysis based on whether they had PLAIN or SWEET first, their sex (male/female), their weight status in the original randomisation (BMI-healthy/BMI-overweight), and their preference for sweet (participants were asked at the end of the study whether they considered themselves to have a ‘sweet tooth’). This randomisation plan for FGF21 was decided due to the growing evidence that FGF21 plays a role in regulating sweet intake and preference (5, 6).

### Controlling for the menstrual cycle

For pre-menopausal women not taking contraceptives, we aimed to allow for the effects of natural monthly fluctuations whilst controlling for these fluctuations in order to make pre- to post-changes comparable. In order to do this, both pre intervention trials (which took fasted measures of health and appetite) were conducted in the same estimate phase of the menstrual cycle, and accordingly, the post-intervention trials were conducted in the same estimated phase. Thus, if different phases of the menstrual cycle did alter appetite or health measures, the pre- to post-changes should still be comparable.

### Specifics of the breakfast intervention

Participants were told that the study was investigating the role of different nutrients and/or flavours at breakfast on health and appetite and that we would be manipulating one or both of these factors. The purpose of intention-masking participants was to somewhat replicate free-living conditions and remove any implicit bias from knowing what the intervention was investigating.

The amount of sugar (30 % by weight) was chosen based on a scoping review of the sugar content of 101 breakfast cereals in the UK in 2017 (**Supplementary Material Table S3**). The average grams of sugar per 100 g of cereal was 19.2 ± 8.1, with the minimum being 0.7 g and the maximum being 37.0 g. Therefore 30 % by weight (i.e. 30 g sugar per 100 g cereal) represented the upper end of what is typically found in breakfast cereals and 7.5 g/100 g higher than the threshold given by the NHS for cereals to be considered as ‘high’ sugar (7). The amount of porridge given was 20 % of the participants resting energy needs measured in the first laboratory visit (detailed below). After the addition of milk, this was estimated to contribute about 20-25 % of participants’ total daily energy needs, with the aim being that this would be sufficient for any effect to be found if there was one, but not so much that participants would struggle to finish the meal. Further, this is approximately in line with public health recommendations which state that breakfast should consist of ~400 kcal (i.e. 20 % energy for a 2000 kcal/d diet) (8).

### Pre-laboratory visit trial standardisation

Prior to the first laboratory visit, participants recorded their exercise for three days, with the aim of the 24-h prior to testing being relatively sedentary (i.e. no vigorous activity). Additionally, participants were asked to record their diet 24-h before coming to the laboratory. During this time, participants were instructed to avoid alcohol, and consume their usual levels of caffeine and nicotine. Food and fluid were prohibited after 2200 h with the exception of plain water of which at least 0.5 L was consumed after waking before coming to the laboratory. Participants were asked to replicate these behaviours before the subsequent three laboratory visits, with the exception that before the two post-intervention laboratory visits, they were asked to consume the breakfast we had provided rather than their original breakfast.

### Laboratory trial standardisation

In the first laboratory visit only, participants underwent an exercise test in order to individually calibrate the activity monitors (ActiHeart™; CamNtech, Cambridge, UK) used during the intervention. Participants wore a heart rate monitor (Polar H10 Heart Rate Sensor, Warwick, UK), and their heart rate and expired gas (as described in the main text) were measured for the final minute of three minutes of exercising on a treadmill at three or four different intensities (dependent upon fitness level and usual exercise patterns). The intensities were: 3.2 km/h, 5.2 km/h, 5.8 km/h at a 10 % upward gradient, and self-selected 9-12 km/h at 0 % gradient (optional, dependent on usual activity levels).

In order to standardize milk within-participant, porridge was made in the laboratory with participants adding the amount of milk they would normally have. The amount of milk added here was then used throughout the intervention to reduce confounding appetitive effects of different volumes of milk. Participants were allowed any type of milk or plant-based milk alternative, providing it did not have any flavour additives (e.g. sweetened soya milk was not allowed, but plain/unsweetened soya milk was), and they used the same milk throughout the intervention. Thus, in both the plain and sweet breakfast trial arms, participants used the same type and amount of milk when making their porridges, based on the amount determined in this laboratory standardization procedure.

### Dietary data analysis

Participants were asked to weigh and record their food and drink and any leftovers. Where weighing items was not possible, participants were given instructions on estimating their portion size, or were instructed to look on food packaging if applicable. Methods of cooking, brands, and names of eating establishments were asked to be recorded in order that these could be used to help in portion size and nutrient information. Three authors (H.A.C., S.R., and P.W) analysed the diet diaries independently (standardized within-participant). Data were analysed using Nutritics online software.

### Physical activity measurement

Physical activity energy expenditure was assessed via ActiHeart™ which uses a two-branch equation based on heart rate and accelerometry. Each ActiHeart was calibrated (as described above) and analysed using ActiHeart™ 4 Software, using the heart rate + stress model. Data were deemed valid for a day based on > 80 % not being “recovered” data (where heart rate signal is poor and energy expenditure being weighted to 75% accelerometry estimates), and based on < 10 % being “lost” data due to non-wear or potential poor contact with the skin. The valid days were then averaged for each trial arm for each participant and used in the analyses. In other words, data were included only for day(s) which met the inclusion criteria (mean valid days = 6; median valid days = 6). No other exclusion criteria were applied (e.g. weekend *versus* weekday).

### Biochemical analysis

The following kits were used for biochemical analyses:

- Serum insulin: Mercodia, product number 10-1113-01
- Plasma GLP-1: Merck Millipore, product number EZGLP1T-36K
- Plasma glucose: RX Daytona, product number GL364, Randox Laboriatories
- Plasma total cholesterol: RX Daytona, product number CH201, Randox Laboriatories
- Plasma TAG: RX Daytona, product number TR212, Randox Laboriatories
- Plasma FGF21: MSD, product number Q9NSA1, Mesoscale Diagnostics LLC
- Serum osmolality: Gonotec Osmomat auto
- Urine specific gravity: SUR-NE Clinical Refractometer, Atago, Japan

Order effectsMost outcomes were not influenced by an order effect. Those that were influenced are outlined in Table S1 below, along with a discussion of the implications of these findings.

# Supplementary Table S1: Outcomes affected by an order effect

| **Outcomes with evidence of an order effect** | | | | |
| --- | --- | --- | --- | --- |
| **Pre- to post-intervention Δ (lab measured) (t-tests/GLM):** | | | | |
|  | **Analysis** | **Original (unadjusted) p-value** | **Adjusted (for order effect) p-value** | **Notes** |
| **Hunger VAS** | Trial*order p = 0.008 | 0.221 | 0.448 | Evidence of order effect but this did not alter the findings |
| **Salt desire VAS** | Trial*order p < 0.001 | 0.888 | 0.013 |  |
| **MEQ** | Trial*order p = 0.021 | 0.164 | 0.011 | Whilst significant, these values were small and did not translate to a change in chronotype category |
| **TFEQ hunger** | Trial*order p = 0.131 | 0.141 | 0.029 | No evidence of order effect, but adjusting for order altered the significance of the finding |
| **Cholesterol** | Trial*order p = 0.234 | 0.194 | 0.035 | No evidence of order effect, but adjusting for order altered the significance of the finding |
| **Across the day during the intervention (ANOVAs, testing trial, time, and trial*time effects):** | | | | |
|  | **Analysis** | **Original (unadjusted) p-value** | **Adjusted (for order effect) p-value** | **Notes** |
| **Fullness VAS** | Trial*order p = 0.868 | Trial p = 0.010 | Trial p = 0.061 | No evidence of order effect, but adjusting for order altered the significance of the finding |
| **How much can you eat VAS** | Trial*time*order p = 0.671 | Trial*time p = 0.014 | Trial*time p = 0.072 | No evidence of order effect, but adjusting for order altered the significance of the finding |
| **Sweet desire VAS** | Trial*order p = 0.049 | Trial p < 0.001 | Trial p < 0.001 | Evidence of order effect but this did not alter the findings |
|  | Trial*time*order p = 0.016 | Trial*time p < 0.001 | Trial*time p = 0.061 |  |
| **Fatty desire VAS** | Trial*order p = 0.045 | Trial p = 0.947 | Trial p = 0.200 | Evidence of order effect but this did not alter the findings |

### Discussion of order effect findings

Order effects appeared to impact nine outcomes in the study. The trend found for each one is discussed below.

**Hunger VAS Δ pre- to post-intervention:** If participants were randomised to PLAIN first, fasted hunger increased by ~7 mm after PLAIN compared to ~2 mm after SWEET. Comparatively, if participants were randomised to SWEET first, hunger decreased by ~4 mm after PLAIN, but increased ~23 mm after SWEET. The original analysis demonstrated Δ+3 mm PLAIN and Δ+11 mm SWEET; thus it might be concluded that when participants consumed SWEET first, their fasted hunger VAS was mitigated by the PLAIN breakfast.

**Salt desire VAS Δ pre- to post-intervention:** If participants were randomised to PLAIN first, fasted salt desire decreased by ~1 mm after PLAIN, and decreased by ~22 mm after SWEET. Comparatively, if participants were randomised to SWEET first, salt desire decreased ~20 mm after PLAIN, but increased ~5 mm after SWEET. The original analysis demonstrated Δ-9 mm PLAIN and Δ-10 mm SWEET; thus it might be concluded that when participants consumed SWEET first, their fasted salt desire VAS was mitigated by the SWEET breakfast.

**MEQ Δ pre- to post-intervention:** If participants were randomised to PLAIN first, MEQ decreased by ~3 after PLAIN, vs increased ~0.5 after SWEET. Comparatively, if participants were randomised to SWEET first, MEQ increased ~0.5 after PLAIN but decreased ~0.8 after SWEET. Whilst this shows a differential effect on raw change scores pre- to post-intervention based on the order of randomisation, these changes did not appear to translate to changes in chronotype categorisation.

**TFEQ hunger Δ pre- to post-intervention:** If participants were randomised to PLAIN first, TFEQ hunger increased by ~0.8 after PLAIN, but decreased ~0.5 after SWEET. Comparatively, if participants were randomised to SWEET first, TFEQ hunger increased by ~0.7 after PLAIN compared to ~0.3 after SWEET. Again, these changes did not seem meaningful considering the scale of TFEQ.

**Cholesterol Δ pre- to post-intervention:** If participants were randomised to PLAIN first, cholesterol decreased by ~0.20 mmol·l^-1^ after PLAIN compared to and increase of ~0.11 mmol·l^-1^ after SWEET. Comparatively, if participants were randomised to SWEET first, cholesterol decreased by ~0.15 mmol·l^-1^ after PLAIN *versus* a decreased of ~0.13 mmol·l^-1^ after SWEET. These results suggest that isocaloric exchange of oats for sugar may result in a slight increase in cholesterol concentrations (i.e. PLAIN first), whereas isocaloric exchange of sugar for oats adds no benefit in terms of cholesterol lowering (i.e. SWEET first). If further research more suited to answering this question confirms such findings, this may have implications for changes in dietary patterns on health, whereby going from low to higher sugar may be more detrimental to health outcomes, whereas going from high to lower sugar may not impact health as notably.

**Fullness VAS across the day:** Whilst there were some slight differences across the day, such as slightly higher fullness pre-lunch after the plain breakfast when participants were randomised to have SWEET first, these differences are small; considering energy balance measures appeared stable, this is unlikely meaningful.

**How much can you eat VAS across the day:** Similar to fullness, these differences appear minimal and unlikely meaningful.

**Sweet desire VAS across the day:** Please see accompanying Figures S1a and S1b. If participants were randomised to PLAIN first, then desire for sweet is consistently higher across the day (ranging from ~25 mm to ~32 mm) after the plain breakfast, with a small (~7 mm) sweet-sensory-specific satiety after breakfast. After the sweet breakfast, a clear sensory-specific satiety of post-breakfast sweet desire is seen, with subsequent sweet desire suppression through the rest of the day. However, sweet desire is also lower pre-breakfast making these results difficult to interpret relative to the plain breakfast.

If participants were randomised to SWEET first, there was no post-breakfast sweet-sensory specific satiety, but a gradual decline (range ~7 mm) in sweet desire across the day after the plain breakfast. After the sweet breakfast, a clear sensory-specific satiety is seen which disappears by lunch.

Overall, the trends are similar; for PLAIN breakfasts, a relative plateau of sweet desire across the day, whereas for SWEET breakfasts, a post-breakfast sensory-specific satiety which somewhat dissipates by lunch. Thus we do not feel this order effect has meaningfully altered our findings.

**Fatty desire VAS across the day:** Similar to fullness, these differences appear minimal (< ~5 mm differences) and unlikely meaningful.


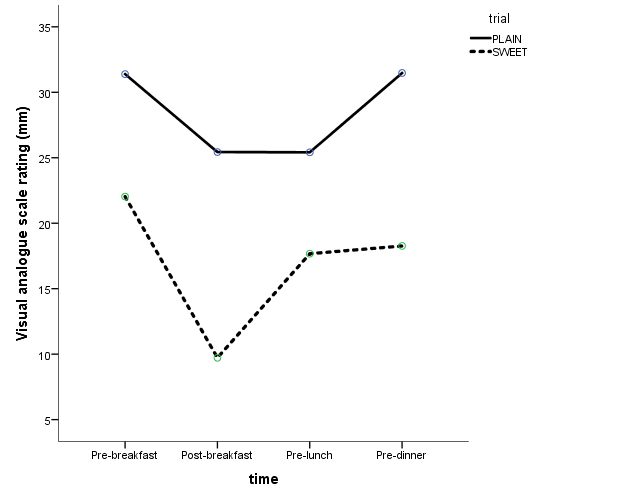


Figure S1a. Visual analogue scale ratings of average sweet desire across the day when participants were randomised to PLAIN first (n = 17)


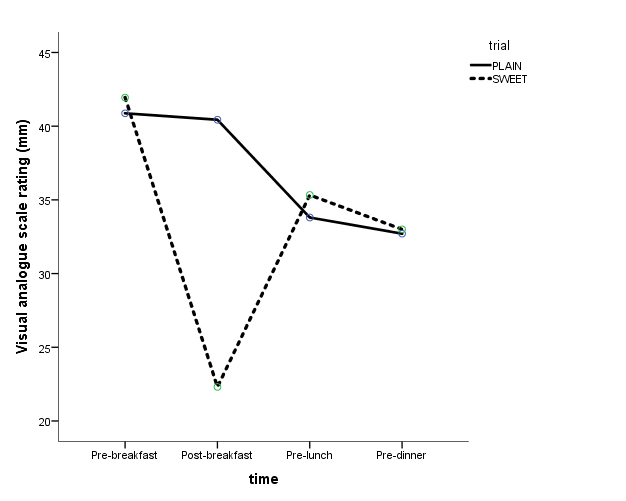


Figure S1b. Visual analogue scale ratings of average sweet desire across the day when participants were randomised to SWEET first (n = 12)

# Supplementary Table S2: Further health and metabolic markers pre- to post-intervention

|  | PLAIN | | | | SWEET | | | |  | | |
| --- | --- | --- | --- | --- | --- | --- | --- | --- | --- | --- | --- |
|  | | Pre  (mean ± *SD*) | Post  (mean ± *SD*) | Δ (95 % CI) pre to post | | Pre  (mean ± *SD*) | Post  (mean ± *SD*) | Δ (95 % CI) pre to post | *p*_diff_ pre PLAIN *vs* SWEET | *p*_diff_ post PLAIN *vs* SWEET | *p*_diff_ Δ PLAIN *vs* SWEET |
| RMR (kcal∙d^-1^)^1^ | | 1513 ± 249 | 1502 ± 270 | -12 (-61, 38) | | 1500 ± 236 | 1529 ± 275 | 29 (-20, 77) | 0.642 | 0.319^a^ | 0.257 |
| RER^1^ | | 0.83 ± 0.05 | 0.84 ± 0.07 | 0.01 (-0.01, 0.02) | | 0.84 ± 0.07 | 0.85 ± 0.05 | 0.00 (-0.03, 0.03) | 0.532^a^ | 0.345^a^ | 0.647^a^ |
| Systolic BP (mmHg) | | 118 ± 9 | 117 ± 10 | -1 (-3, 2) | | 116 ± 10 | 118 ± 10 | 2 (0, 4) | 0.128 | 0.438^a^ | 0.099 |
| Diastolic BP (mmHG) | | 76 ± 6 | 77 ± 6 | 1 (-1, 2) | | 76 ± 6 | 78 ± 7 | 2 (0, 4) | 0.646 | 0.220 | 0.266 |
| Urine specific gravity | | 1.013 ± 0.008 | 1.013 ± 0.008 | 0.001 (-0.002, 0.004) | | 1.014 ± 0.009 | 0.012 ± 0.008 | -0.002 (-0.004, 0.001) | 0.286 | 0.222 | 0.061^a^ |
| Sosm (mOsm∙kg^-1^)^4^ | | 296 ± 6 | 296 ± 6 | 0 (-2, 3) | | 295 ± 5 | 296 ± 4 | 1 (-1, 4) | 0.209 | 0.912 | 0.418 |

Abbreviations: BP, blood pressure; RER, respiratory exchange ratio; RMR, resting metabolic rate; *SD*, standard deviation; Sosm, serum osmolality

n = 29 for systolic BP, diastolic BP, urine specific gravity; n = 27 for RMR and RER; n = 23 for Sosm

^a^Data analysed using Wilcoxon signed rank test. All other data analysed using paired samples t-test

# Supplementary Table S3: Psychological appetite changes pre- to post- each intervention (n = 29)

|  | PLAIN | | | | SWEET | | | | |  | | |
| --- | --- | --- | --- | --- | --- | --- | --- | --- | --- | --- | --- | --- |
|  | | Pre  (mean ± *SD*) | Post  (mean ± *SD*) | Δ (95 % CI) pre to post | | Pre  (mean ± *SD*) | Post  (mean ± *SD*) | Δ (95 % CI) pre to post | *p*_diff_ pre PLAIN *vs* SWEET | | *p*_diff_ post PLAIN *vs* SWEET | *p*_diff_ Δ PLAIN *vs* SWEET |
| VAS hunger (mm)^a^ | | 55 ± 24 | 58 ± 23 | 3 (-6, 11) | | 49 ± 24 | 59 ± 23 | 11 (1, 20) | 0.176 | | 0.726 | 0.221 |
| VAS fullness (mm)^a^ | | 26 ± 22 | 27 ± 21 | 0 (-7, 8) | | 26 ± 23 | 23 ± 17 | -3 (-9, 3) | 0.962 | | 0.296^b^ | 0.897^b^ |
| VAS eat (mm)^a^ | | 62 ± 17 | 62 ± 20 | 0 (-7, 7) | | 58 ± 21 | 62 ± 18 | 4 (-3, 12) | 0.220 | | 0.952 | 0.356^b^ |
| VAS thirst (mm)^a^ | | 54 ± 24 | 45 ± 27 | -9 (-19, 1) | | 53 ± 23 | 52 ± 25 | -1 (-9, 7) | 0.906 | | 0.067 | 0.220 |
| VAS sweet (mm)^a^ | | 41 ± 25 | 36 ± 26 | -5 (-13, 3) | | 40 ± 23 | 31 ± 24 | -8 (-17, 0) | 0.938 | | 0.215 | 0.459 |
| VAS salt (mm)^a^ | | 35 ± 21 | 25 ± 22 | -9 (-20, 2) | | 39 ± 24 | 29 ± 24 | -10 (-20, 1) | 0.364 | | 0.396 | 0.888 |
| VAS savoury (mm)^a^ | | 49 ± 26 | 46 ± 28 | -3 (-11, 5) | | 54 ± 24 | 43 ± 26 | -10 (-17, -4) | 0.199 | | 0.470 | 0.152 |
| VAS fatty (mm)^a^ | | 25 ± 23 | 23 ± 24 | -2 (-9, 4) | | 26 ± 24 | 25 ± 26 | -1 (-9, 7) | 0.639^b^ | | 0.477^b^ | 0.762 |
| FC trait | | 117 ± 30 | 116 ± 31 | -1 (-5, 3) | | 117 ± 35 | 115 ± 32 | -7 (-17, 3) | 0.449^b^ | | 0.672 | 0.885^b^ |
| FC state | | 42 ± 11 | 43 ± 12 | 0 (-3, 4) | | 43 ± 10 | 44 ± 11 | 1 (-2, 4) | 0.890 | | 0.493 | 0.766 |
| FCI fatty | | 12 ± 4 | 12 ± 3 | 0 (-2, 1) | | 13 ± 4 | 12 ± 5 | -1 (-1, 0) | 0.329 | | 0.554 | 0.750 |
| FCI sweet | | 15 ± 4 | 15 ± 5 | 0 (-1, 2) | | 16 ± 6 | 16 ± 5 | -1 (-2, 1) | 0.464 | | 0.921 | 0.535 |
| FCI CHO | | 15 ± 5 | 14 ± 4 | -1 (-2, 1) | | 15 ± 5 | 15 ± 4 | 0 (-2, 1) | 0.572 | | 0.233 | 0.704 |
| FCI fast food | | 8 ± 3 | 9 ± 4 | 0 (-1, 2) | | 9 ± 3 | 8 ± 3 | -1 (-1, 0) | 0.444 | | 0.332 | 0.253 |
| TFEQ restraint | | 10 ± 5 | 9 ± 5 | -1 (-2, 0) | | 10 ± 5 | 9 ± 5 | 0 (-1, 1) | 0.141 | | 0.628 | 0.446 |
| TFEQ disinhibition | | 8 ± 4 | 7 ± 4 | 0 (-1, 0) | | 7 ± 3 | 8 ± 3 | 0 (-1, 0) | 0.068^b^ | | 0.489 | 0.630 |
| TFEQ hunger | | 6 ± 4 | 7 ± 4 | 1 (0, 1) | | 7 ± 3 | 7 ± 3 | 0 (-1, 0) | 0.056 | | 0.907 | 0.141 |
| MEQ | | 53 ± 9 | 52 ± 10 | -1 (-3, 0) | | 53 ± 8 | 52 ± 9 | 0 (-1, 1) | 0.501 | | 0.515^b^ | 0.164 |

Abbreviations: FC, Food Craving; FCI, Food Craving Inventory; MEQ, Morningness-Eveningness Questionnaire; TFEQ, Three Factor Eating Questionnaire; VAS, visual analogue scales

^a^VAS questions: “How hungry do you feel?”, “How full do you feel?”, “How much food do you think you can eat?”, “How thirsty do you feel?”, “How strong is your desire to eat something sweet?”, “How strong is your desire to eat something savoury?”, “How strong is your desire to eat something salty?”, “How strong is your desire to eat something fatty?”

^b^Data analysed using Wilcoxon signed rank test. All other data analysed using paired samples t-test

# Supplementary Material Table S4: Review of sugar content of 101 UK breakfast cereals

| **Brand** | **Cereal** |  | **kcal/100 g** | **Fat**  **g /100 g** | **Saturates g/100 g** | **Carbs g/100 g** | **Sugar g/100 g** | **Fibre**  **g/100 g** | **Protein g/100 g** |
| --- | --- | --- | --- | --- | --- | --- | --- | --- | --- |
| Quaker | Oats so simple original porridge |  | 370 | 7.7 | 1.3 | 58.9 | 1 | 10.5 | 11 |
|  | Oats so simple golden syrup porridge |  | 376 | 6.5 | 1.1 | 66.6 | 21.5 | 8.8 | 8.4 |
|  | Porridge oats |  | 374 | 8 | 1.5 | 60 | 1.1 | 9 | 11 |
|  | Oats so simple sultana, raisin, cranberry and apple porridge |  | 371 | 5.7 | 0.9 | 68.2 | 26.2 | 6.9 | 8.3 |
|  | Oats so simple apple and blueberry porridge |  | 376 | 6.4 | 1.1 | 67.1 | 20.9 | 7.7 | 8.6 |
|  | Oats so simple sweet cinnamon porridge |  | 376 | 5.2 | 1.1 | 65 | 28.4 | 5.6 | 14.4 |
|  | Oat crisp |  | 375 | 6.5 | 1 | 58 | 10 | 15 | 13 |
|  | Oat so simple summer berries porridge |  | 375 | 4.7 | 0.8 | 66.6 | 26.4 | 4.4 | 14.3 |
|  | Oats so simple honey and vanilla |  | 381 | 6.4 | 1.2 | 68.1 | 18.8 | 7.2 | 8.8 |
|  | Oat granola raisin |  | 418 | 9.2 | 1.2 | 70.9 | 22.5 | 6.8 | 8 |
|  | Oat so simple apple and cherry porridge |  | 375 | 6.1 | 1.2 | 68 | 22.3 | 7.5 | 8.5 |
|  | Super goodness apple, cinnamon, and raisin porridge |  | 373 | 7.4 | 1.3 | 63.7 | 18.6 | 8.5 | 11.5 |
|  | Oat so simple maple and pecan porridge |  | 376 | 6.5 | 1.1 | 66.6 | 21.5 | 8.8 | 8.4 |
|  | Oat so simple strawberry jam porridge |  | 378 | 6.5 | 1.2 | 67.3 | 19 | 7.3 | 8.9 |
|  | Oat muesli, nuts and seeds |  | 393 | 12 | 1.4 | 66 | 12.4 | 9.5 | 10.8 |
| Weetabix | Original |  | 362 | 2 | 0.6 | 69 | 4.4 | 10 | 12 |
|  | Banana |  | 357 | 2 | 0.4 | 70 | 15 | 9.7 | 10 |
|  | Minis chocolate |  | 391 | 5.4 | 2.6 | 71 | 21 | 9.4 | 9.8 |
|  | Oatibix golden oats flakes |  | 399 | 6.5 | 1 | 72 | 14 | 7.2 | 9.5 |
|  | Chocolate |  | 368 | 4 | 1.7 | 68 | 18 | 10 | 10 |
|  | Oatibix |  | 395 | 8 | 1.3 | 64 | 3.2 | 7.3 | 13 |
|  | Crunchy bran |  | 352 | 3.6 | 0.6 | 58 | 14 | 20 | 12 |
|  | Minis fruit and nut |  | 371 | 4.1 | 0.6 | 69 | 22 | 9.3 | 9.9 |
|  | Baked with golden syrup |  | 362 | 1.9 | 0.4 | 72 | 15 | 8.2 | 10 |
|  | Protein |  | 360 | 1.9 | 0.4 | 62 | 4.8 | 9.6 | 19 |
|  | Oatibix flakes mixed raisin and sultana |  | 381 | 4.9 | 0.9 | 73.9 | 24.9 | 5.6 | 7.4 |
|  | Minis banana |  | 389 | 4.9 | 3 | 72 | 20 | 9.7 | 9.3 |
|  | Additions coconut and raisins |  | 363 | 5.7 | 4 | 62 | 15 | 13 | 9.5 |
|  | Additions apple and raisin |  | 344 | 1.8 | 0.4 | 66 | 15 | 13 | 9.5 |
|  | Wheetos chocolatey |  | 388 | 4.6 | 0.9 | 75 | 21 | 6.5 | 8.5 |
| Kellogg's | Coco pops |  | 387 | 2.5 | 1 | 84 | 30 | 3 | 5.5 |
|  | Fruit 'n' fibre cereal |  | 380 | 6 | 3.5 | 69 | 24 | 9 | 8 |
|  | Just right cereal |  | 371 | 2 | 0.3 | 79 | 23 | 4.5 | 7 |
|  | Start cereal |  | 390 | 3.5 | 2 | 79 | 24 | 5 | 8 |
|  | Crunchy nut cornflakes |  | 398 | 4.5 | 0.7 | 82 | 35 | 2.5 | 6 |
|  | Disney Moana honey multigrain cereal |  | 379 | 2.5 | 0.5 | 77 | 21 | 8 | 8 |
|  | Cornflakes |  | 378 | 0.9 | 0.2 | 84 | 8 | 3 | 7 |
|  | Rice krispies |  | 383 | 1 | 0.2 | 87 | 10 | 1 | 6 |
|  | Rice krispies multigrain |  | 379 | 2.5 | 0.5 | 77 | 21 | 8 | 8 |
|  | Special K |  | 375 | 1.5 | 0.3 | 79 | 15 | 4.5 | 9 |
|  | All bran flakes |  | 359 | 3.2 | 0.5 | 63 | 14 | 15 | 12 |
|  | Frosties |  | 375 | 0.6 | 0.1 | 87 | 37 | 2 | 4.5 |
|  | Special K red berries |  | 376 | 1.5 | 0.3 | 79 | 17 | 5.3 | 8.9 |
|  | Crunchy nut clusters chocolate |  | 460 | 17 | 5 | 67 | 31 | 3.5 | 8 |
|  | Krave milk chocolate |  | 441 | 14 | 4 | 70 | 28 | 3.1 | 7.2 |
|  | All bran |  | 334 | 3.5 | 0.7 | 48 | 18 | 27 | 14 |
|  | Krave chocolate hazelnut |  | 450 | 16 | 4 | 68 | 29 | 3.3 | 6.9 |
|  | Bran flakes |  | 359 | 3.2 | 0.5 | 63 | 14 | 15 | 12 |
|  | Crunchy nut clusters honey and nut |  | 447 | 15 | 3 | 68 | 26 | 4 | 8 |
|  | All bran golden crunch |  | 405 | 11 | 1.5 | 62 | 21 | 13 | 8 |
|  | Crunchy nut bites, nut and caramel |  | 450 | 15 | 7 | 71 | 27 | 3.5 | 6 |
|  | Ricicles |  | 383 | 0.8 | 0.1 | 89 | 34 | 0.8 | 4.5 |
|  | Saltana bran |  | 350 | 2.4 | 0.4 | 67 | 30 | 11 | 9.6 |
|  | Crunchy nut granola, chocolate and nut |  | 497 | 25 | 12 | 57 | 21 | 5.8 | 8 |
|  | Special K peach and apricot |  | 373 | 1.5 | 0.3 | 79 | 19 | 4.5 | 8 |
|  | Special K oats and honey |  | 381 | 3 | 0.5 | 77 | 18 | 5 | 9 |
|  | Special K fruit and nut |  | 372 | 2.5 | 0.4 | 78 | 27 | 4.4 | 7.4 |
|  | Crunch nut granola, fruit and nut |  | 477 | 21 | 8.7 | 63 | 29 | 4.6 | 6.8 |
|  | Special K chocolate flakes |  | 396 | 5.5 | 2.8 | 76 | 22 | 4.1 | 8.6 |
|  | Coco pops porridge |  | 397 | 6.9 | 1.2 | 70 | 20 | 8.5 | 9.4 |
|  | Wheat's, frosted |  | 364 | 2 | 0.6 | 72 | 17 | 9 | 10 |
|  | Wheat's, raisin |  | 345 | 2 | 0.4 | 69 | 13 | 9 | 9 |
|  | Ancient legends granola rye, chia seeds, saltuna and pumpkin seeds |  | 427 | 16 | 5.4 | 56 | 20 | 9.3 | 10 |
|  | Ancient legends granola cranberry and seeds |  | 409 | 12 | 4.2 | 63 | 28 | 8.3 | 8.2 |
|  | Crunchy nut porridge |  | 415 | 9.3 | 1.7 | 70 | 20 | 6.3 | 9.7 |
|  | Crunchy nut peanut butter |  | 488 | 25 | 6.7 | 49 | 12 | 5.5 | 14 |
|  | Granola, delightful fruit |  | 398 | 9.1 | 2.5 | 65 | 20 | 10 | 8.9 |
|  | Coco pops granola |  | 451 | 17 | 2.5 | 65 | 18 | 4.8 | 7.2 |
|  | Muesli, delightful fruit |  | 369 | 5.1 | 0.8 | 67 | 18 | 9.1 | 9.3 |
|  | Special K blueberries, blackberries, raspberries and recurrants |  | 374 | 3.4 | 0.5 | 71 | 14 | 9.6 | 10 |
|  | Special K nourish flakes, cluster and seeds cranberry, red apple and pumpkin seeds |  | 381 | 4.8 | 0.8 | 70 | 17 | 8.9 | 10 |
|  | Granola, glorious nut |  | 420 | 12 | 2.6 | 63 | 17 | 9.9 | 10 |
|  | Special K nourish flakes, cluster and seeds hazelnuts, almonds and pumpkin seeds |  | 404 | 9.6 | 0.7 | 63 | 12 | 8.6 | 12 |
| Nestle | Cheerios |  | 378 | 3.7 | 0.8 | 74 | 21 | 7.8 | 8.8 |
|  | Shreddies |  | 364 | 1.7 | 0.3 | 71 | 15 | 12 | 11 |
|  | Shredded wheat |  | 362 | 2.2 | 0.5 | 68 | 0.7 | 12 | 12 |
|  | Curiously cinnamon cereal |  | 418 | 10 | 4.3 | 47 | 25 | 5.2 | 5.7 |
|  | Coco shreddies |  | 369 | 1.9 | 0.6 | 74 | 28 | 11 | 9 |
|  | Shredded wheat bitesize |  | 367 | 2.2 | 0.5 | 69 | 0.7 | 13 | 12 |
|  | Frosted shreddies |  | 368 | 1.5 | 0.2 | 75 | 27 | 10 | 9 |
|  | Cheerios honey |  | 378 | 3.3 | 0.6 | 75 | 24 | 7.5 | 8.4 |
|  | Cookie crisp |  | 382 | 3.3 | 1.4 | 78 | 25 | 5.7 | 6.7 |
|  | Shredded wheat honey nut cereal |  | 387 | 5.4 | 1.2 | 68 | 15 | 9.8 | 12 |
|  | Golden nuggets |  | 378 | 1.5 | 0.4 | 81 | 25 | 5 | 7.5 |
|  | Oats and more almond cereal |  | 404 | 8.8 | 1.3 | 67 | 25 | 7 | 11 |
|  | Nesquik chocolate cereal |  | 381 | 4 | 1.8 | 74 | 25 | 8.7 | 7.8 |
|  | Shreddies honey |  | 372 | 1.8 | 0.2 | 75.9 | 26.9 | 8.8 | 8.8 |
|  | Oats and more raisin cereal |  | 373 | 4.8 | 0.9 | 69 | 29 | 8.1 | 8.4 |
|  | Clusters cereal |  | 377 | 3.4 | 1.3 | 74 | 22 | 7.9 | 9 |
|  | Golden grahams |  | 385 | 3.1 | 1.1 | 80 | 25 | 4.7 | 7 |
|  | Cheerios chocolatey |  | 385 | 5 | 1.1 | 73 | 25 | 7.8 | 8.4 |
|  | Lion chocolate cereal |  | 412 | 7.3 | 76 | 28.8 | 7.6 | 3 | 5 |
|  | Cheerios oat crisp original |  | 385 | 5.2 | 0.9 | 70 | 23 | 8.5 | 11 |
|  | Cheerios aot crisp cinnamon |  | 385 | 5.2 | 0.9 | 70 | 23 | 8.5 | 11 |
|  | Shreddies max oat granola cereal |  | 403 | 7.1 | 0.8 | 68 | 23 | 7.3 | 13 |
|  | Shreddies max cranberry and oat granola cereal |  | 403 | 7 | 0.8 | 68 | 24 | 7.2 | 13 |
|  | Shredded wheat multigrain barley and spelt |  | 373 | 2.4 | 0.5 | 72 | 15 | 10 | 11 |
|  | Shredded wheat multigrain rye and quinoa |  | 373 | 2.5 | 0.5 | 72 | 15 | 10 | 11 |
| Scott's | Original porage oats |  | 374 | 8 | 1.5 | 60 | 1 | 9 | 11 |
|  | Old fashioned porage oats |  | 374 | 8 | 1.5 | 60 | 1.1 | 9 | 11 |
|  | So-easy porage original porridge oats |  | 379 | 8.5 | 1.5 | 60 | 1 | 9 | 11 |

Mean sugar content: 19.2 g/100 g

Median sugar content: 20.9 g/100 g


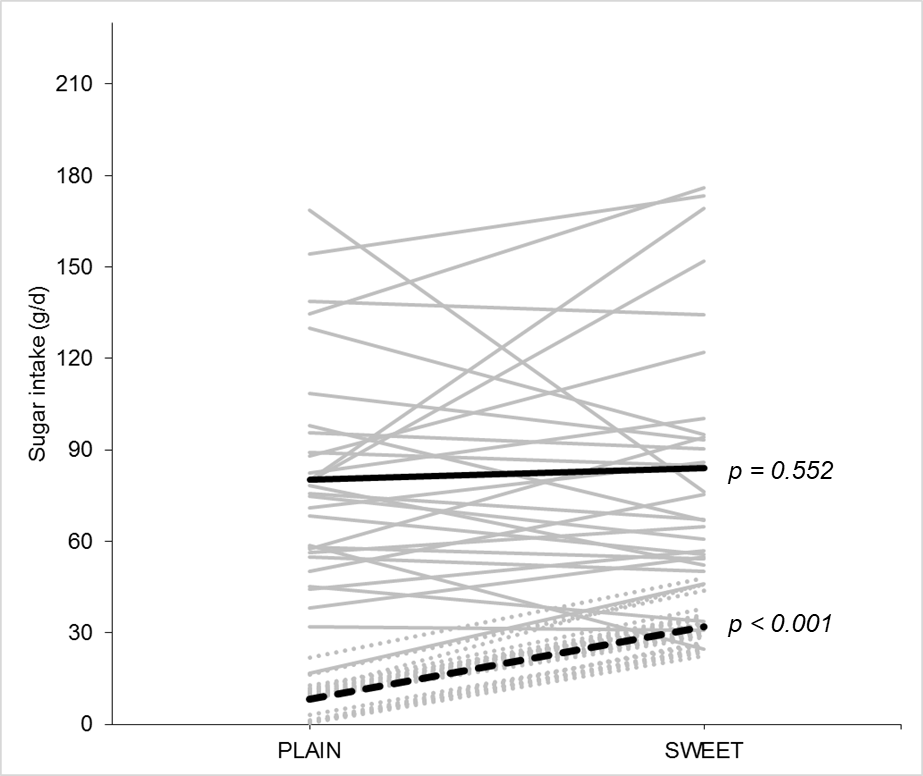


# Supplementary Figure S2: Individual average post-breakfast (solid lines) and breakfast (dashed lines) sugar intake per day (n = 29)


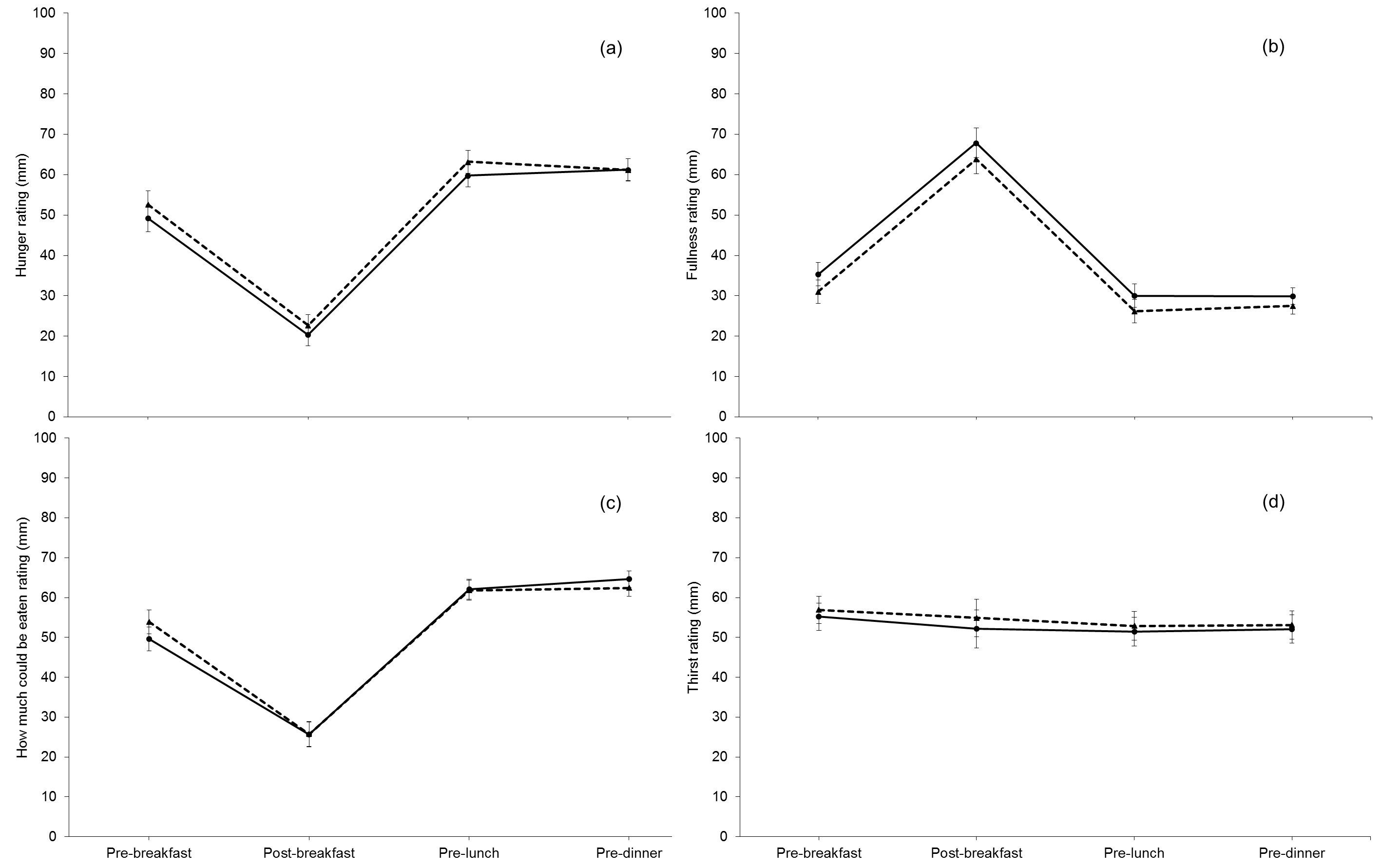


# Supplementary Figure S3: Visual analogue scale ratings for hunger (a), fullness (b), satiety (c), and thirst (d) across the day (n = 29)

Dashed lines SWEET, full lines, PLAIN. Error bars: normalised confidence intervals

# Further discussion points

## Cholesterol

There were no differences in the change pre- to post-intervention in total plasma cholesterol concentrations, but we did find a small reduction in plasma total cholesterol concentration after PLAIN but not SWEET. The reduction after PLAIN of -0.19 mmol∙l-1 is in line with other studies feeding oat β-glucan (9). This could be due to the higher oat content of the PLAIN porridge, though fibre was only ~2 g different between trial arms; it is unclear if this difference in fibre would be meaningful in terms of lowering cholesterol. Alternatively, these findings may suggest that the addition of sugar may mitigate the known cholesterol-lowering effects of oat β-glucan; this hypothesis certainly warrants testing since our study or analysis was not designed to make these inferences.

## Palatability

Higher palatability has also been shown to increase energy intake (10, 11) but this may have been negated as liking of both the PLAIN and SWEET breakfasts were similar. Slightly lower desire for sweet was found pre-dinner which may be due to an order effect whereby participants had overall lower desire for sweet during their second trial arm. Despite this, there was no evidence that the post-breakfast reduction in sweet desire was the result of an order effect.

# Supplementary Material References

1. Lovejoy JC, Sainsbury A, Stock Conference Working G. Sex differences in obesity and the regulation of energy homeostasis. Obesity reviews : an official journal of the International Association for the Study of Obesity. 2009;10(2):154-67.

2. Westerterp KR, Meijer GA, Janssen EM, Saris WH, Ten Hoor F. Long-term effect of physical activity on energy balance and body composition. The British journal of nutrition. 1992;68(1):21-30.

3. Betts JA, Richardson JD, Chowdhury EA, Holman GD, Tsintzas K, Thompson D. The causal role of breakfast in energy balance and health: a randomized controlled trial in lean adults. The American journal of clinical nutrition. 2014;100(2):539-47.

4. Hetherington MM, Pirie LM, Nabb S. Stimulus satiation: effects of repeated exposure to foods on pleasantness and intake. Appetite. 2002;38(1):19-28.

5. Soberg S, Sandholt CH, Jespersen NZ, Toft U, Madsen AL, von Holstein-Rathlou S, et al. FGF21 Is a Sugar-Induced Hormone Associated with Sweet Intake and Preference in Humans. Cell Metab. 2017;25(5):1045-53 e6.

6. Talukdar S, Owen BM, Song P, Hernandez G, Zhang Y, Zhou Y, et al. FGF21 Regulates Sweet and Alcohol Preference. Cell Metab. 2016;23(2):344-9.

7. NHS Eat Well. Healthy Breakfast Cereals 2017 [Available from: https://www.nhs.uk/live-well/eat-well/healthy-breakfast-cereals/.

8. National Health Service. Healthy Breakfast Cereals 2017 [Available from: https://www.nhs.uk/live-well/eat-well/healthy-breakfast-cereals/.

9. Whitehead A, Beck EJ, Tosh S, Wolever TM. Cholesterol-lowering effects of oat beta-glucan: a meta-analysis of randomized controlled trials. The American journal of clinical nutrition. 2014;100(6):1413-21. doi: 10.3945/ajcn.114.086108. PubMed PMID: 25411276; PubMed Central PMCID: PMCPMC5394769.

10. Monneuse MO, Bellisle F, Louis-Sylverstre J. Responses to an intense sweetener in humans: immediate preference and delayed effects on intake. Physiology & behavior. 1991;49(2):325-30. PubMed PMID: 2062905.

11. Perez C, Dalix AM, Guy-Grand B, Bellisle F. Human responses to five concentrations of sucrose in a dairy product: immediate and delayed palatability effects. Appetite. 1994;23(2):165-78. doi: 10.1006/appe.1994.1044. PubMed PMID: 7864610.
